# Common Bacterial Isolates Associated With Neonatal Sepsis and Their Antimicrobial Profile: A Retrospective Study at King Abdulaziz University Hospital, Jeddah, Saudi Arabia

**DOI:** 10.7759/cureus.21107

**Published:** 2022-01-11

**Authors:** Azzah S Alharbi

**Affiliations:** 1 Medical Microbiology and Parasitology, Faculty of Medicine, King Abdulaziz University, Jeddah, SAU; 2 Special Infectious Agents Unit, King Fahd Medical Research Center, King Abdulaziz University, Jeddah, SAU

**Keywords:** neonates, septicaemia, microbial pathogens, infection, antibiotic sensitivity

## Abstract

Background

Neonatal sepsis is a major contributor to morbidity and mortality among neonates. There has been considerable geographic variation in causative pathogens and antimicrobial sensitivity profiles over time. This makes the continuous monitoring of patterns of emergence crucial for the effective implementation of antimicrobial therapy guidelines in an attempt to control antimicrobial resistance.

Methods

A retrospective study was conducted among neonates with sepsis admitted to King Abdulaziz University Hospital, Jeddah, Saudi Arabia, between May 2011 and October 2018. The data were collected from medical records in the neonatal intensive care unit and analyzed using SPSS version 20 (IBM Corp., Armonk, NY).

Results

There were 246 neonates clinically diagnosed with sepsis, of whom 40 (16.26%) had positive blood cultures. In the blood cultures, coagulase-negative *Staphylococcus* was the most prevalent microorganism (57.5%), followed by *Klebsiella *spp. (10%). *Streptococcus agalactiae*, *Enterobacter cloacae*, *Escherichia coli*, *Acinetobacter baumanii*, and *Candida* spp. each accounted for 5% of all isolates. Only single isolates of methicillin-resistant *Staphylococcus aureus*, *Pseudomonas aeruginosa,* and *Bacillus *spp. (2.5% each) were detected in this study. Most of the isolated microorganisms exhibited high sensitivity to ampicillin and gentamicin.

Conclusions

This study points to a likely emergence of coagulase-negative *Staphylococci* as the main cause of sepsis among neonates. Ampicillin and gentamicin are highly effective against the commonly isolated bacterial pathogens that cause neonatal sepsis.

## Introduction

Neonatal sepsis, i.e., any sepsis determined in the first 28 days after birth, is widely prevalent and continues to be a substantial contributor to newborn mortality worldwide [1-3]. Although the past two decades have seen an increase in published studies addressing the pathomechanism of sepsis and its therapeutic strategies, the most recent statistics reveal that sepsis-related neonatal mortality has not significantly improved. Globally, an estimated one million annual newborn deaths are due to neonatal sepsis [4]. In middle and low-income countries, 30%-50% of all neonatal deaths are related to sepsis [5]. Indeed, sepsis-related mortality is largely preventable with timely diagnosis and rapid treatment using effective antimicrobial agents [6]. Unfortunately, there is a lack of rapid, robust diagnostic methods to detect the invading pathogens that cause sepsis [7]. The current “gold standard” diagnostic method, blood culture, has variable sensitivity (50%-80%) and can take hours to days to yield results [8]. Thus, the initiation of empirical antimicrobial therapy for suspected sepsis cases is essential [9]. Identifying the prevalent pathogens that cause neonatal sepsis and their sensitivity pattern will aid physicians in effectively initiating empirical treatment for such cases with the appropriate antimicrobial agents.

However, the pathogens that cause neonatal sepsis differ across geographical locations, even in the same country, and their pattern changes continuously over time. Moreover, many of them have developed increased resistance to the wide range of commonly used antimicrobial agents, making treatment extremely difficult [10-11]; their antimicrobial resistance pattern also varies from one geographical area to another [12-13]. Periodic surveillance of the causative organisms and their antimicrobial susceptibility profile is potentially valuable for updating the empirical therapeutic strategy for suspected sepsis with effective and targeted antimicrobial agents and, thus, could limit antimicrobial resistance, the current major challenge in sepsis management. While this crucial information is regularly monitored and updated in health care settings in developed countries and some developing countries, relevant updated data from different regions in Saudi Arabia is scarce. Before 2000, *Staphylococcus epidermidis* was reported as the most common pathogen isolated from neonates with sepsis admitted to tertiary care hospitals in Riyadh [14] and Khobar [15] retrospectively. Another retrospective analysis of sepsis reported a similar finding in neonates with low birth weight (500 and 1500 grams) admitted to King Khalid University Hospital in Riyadh from 1999 through 2007 [16]. Group B *Streptococcus* and *Escherichia coli *were frequently recovered from septic infants hospitalized at King Fahad Medical City in Riyadh from 2011 to 2015 [17]. To our knowledge, the local microbial pattern of neonatal sepsis in the Western Region of Saudi Arabia has not yet been documented. This study aimed to assess the causative microorganisms of neonatal sepsis and their antimicrobial susceptibility profile in the neonatal intensive care unit (NICU) at King Abdulaziz University Hospital (KAU Hospital) in Jeddah.

## Materials and methods

A retrospective study was conducted using information from the NICU and microbiology department database in King Abdulaziz University Hospital, Jeddah, Saudi Arabia. The study received ethical approval from the institutional review board of the KAU Hospital (IRB approval number 641-19). The records available between May 2011 and October 2018 were reviewed during the study period. All neonates (0-28 days of age) born in or admitted to the hospital with clinically diagnosed sepsis and who had a blood culture test were included in this study. Sepsis was clinically diagnosed if the infant presented with signs and symptoms of systemic inflammatory response syndrome (SIRS) attributed to microbial etiology whether confirmed microbiologically or not. Blood samples were collected and processed following standard microbiological techniques. Prior to the initiation of empirical antimicrobial therapy, one to three milliliters of venous blood sample was aseptically drawn into sterile blood culture vials. Specimens were instantly delivered to the medical microbiology laboratory and incubated at room temperature for not less than 24 hours. Specimens with signs of growth were further processed by sub-culturing and other microbial identification methods [18]. Antibiotic susceptibility of the microbial isolates was tested by the Kirby Bauer disc diffusion method and the results were interpreted in accordance with the National Committee for Clinical Laboratory Standards [19]. Resistance to more than two classes of antimicrobial agents was regarded as a multidrug-resistant pathogen (MDR) [20]. The sensitivity of specific microbial isolates to each tested antimicrobial agent was estimated as a percentage by dividing the number of susceptible isolates by the total number of isolates. Data about sex, blood culture, and antibiotic susceptibility profiles were recorded, coded, and entered into a Microsoft Excel sheet (Microsoft Corporation, Redmond, WA). Statistical analysis was performed using SPSS version 20 (IBM Corp., Armonk, NY). Data were presented as frequency, percentages, and a simple pie chart.

## Results

**Figure 1:**
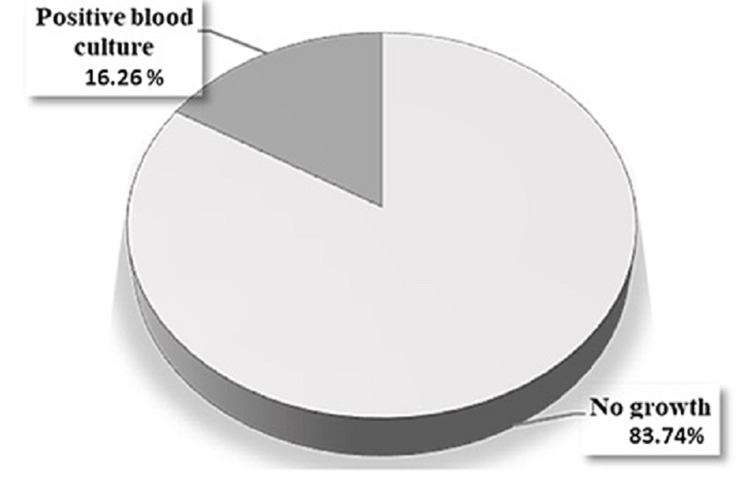
Distribution of neonatal sepsis cases according to blood culture confirmation

A total of 246 neonates admitted to the NICU with clinically diagnosed sepsis were included in this study; 58.75% (n=141) were male and 43.75% (n=105) were female. As indicated in Figure 1, only 40 neonates with sepsis were confirmed positive by blood culture, of which 47.6% (n=19) were in males and 52.4% (n=21) were in females. The remaining septic neonates cases (n=206) had negative blood cultures. The correlation between sex and the positivity of blood culture was not significant (P>0.05). Slightly more than two-thirds of the total recovered isolates were gram-positive (67.5%, n=27) while gram-negative isolates (27.5%, n=11) and fungi (5%, n=2) accounted for the remaining third. Coagulase-negative *Staphylococcus* (CoNS) was the most commonly isolated microorganism (57.5%), followed by *Klebsiella spp.* (10%). Each of the following pathogens accounted for 5% of all isolates: *Streptococcus agalactia*,* Enterobacter cloacae*,* Escherichia coli*,* Acinetobacter baumannii*, and *Candida spp.* Only single isolates of methicillin-resistant *Staphylococcus aureus* (MRSA), *Pseudomonas aeruginosa*, and *Bacillus spp.* (2.5% each) were detected in this study (Table 1). Isolated contaminants recognized by the microbiologist were excluded.

**Table 1:** Microbial isolates from blood cultures Spp.: species Data are presented as numbers and percentages.

Microbial isolates		Count	Percentage
Gram-positive microorganism	Coagulase-negative Staphylococcus	23	57.5%
	Methicillin-resistant Staphylococcus aureus (MRSA)	1	2.5%
	Streptococcus agalactiae	2	5%
	Bacillus spp.	1	2.5%
	Subtotal	27	67.5%
Gram-negative microorganism	Klebsiella spp.	4	10%
	Escherichia coli	2	5%
	Pseudomonas aeruginosa	1	2.5%
	Enterobacter cloacae	2	5%
	Acinetobacter baumanii	2	5%
	Subtotal	11	27.5%
Fungi	Candida spp.	2	5%
Total		40	100 %

In this study, ampicillin was found to be the most effective antibiotic against CoNS isolates (91.3%, 21/23), followed by gentamicin (82.6%, 19/23). Cefotaxime, cloxacillin, and amikacin showed low-to-moderate effects, with sensitivity rates of 56.52% (13/23), 47.8% (11/23), and 39.13% (9/23), respectively. Only 26% (6/23) and 17.4% (4/23) of CoNS isolates were sensitive to piperacillin + tazobactam and meropenem, respectively. The least effective antimicrobial agent was ceftriaxone, with a sensitivity rate of 6.25% (2/23). Of *Klebsiella spp.*, 75%-100% were sensitive to cloxacillin, gentamycin, amikacin, and meropenem; however, 75% (3/4) were resistant to ceftriaxone. *Streptococcus agalactiae* showed 100% (2/2) sensitivity to ampicillin and cefotaxime and 50% (1/2) resistance to gentamicin. All *Enterobacter cloacae* isolates (100%, 2/2) were sensitive to ampicillin, gentamycin, amikacin, and erythromycin while only 50% (1/2) were sensitive to cloxacillin, ceftriaxone, meropenem, and piperacillin + tazobactam. The sensitivity rate of *Escherichia coli* was 100% (2/2) to ampicillin, cloxacillin, gentamycin, amikacin, and cefotaxime and 50% (1/2) to ceftriaxone and meropenem. Of *Acinetobacter baumannii* isolates, one of two (50%) was sensitive to ampicillin, amikacin, cefotaxime, and meropenem while 100% (2/2) showed sensitivity to gentamycin alone. MRSA was sensitive to cloxacillin, cefotaxime, amikacin, and meropenem. One-hundred percent of both Pseudomonas aeruginosa and Bacillus spp. isolates were sensitive to ampicillin, gentamycin, cloxacillin, and amikacin (Table 2).

**Table 2:** Relative sensitivity pattern of microbial isolates to antimicrobial agents S/T: Number of sensitive organisms/total; pip-tazo: piperacillin-tazobactam; ND; not documented; Spp.: species Data are presented as numbers and percentages.

	Antimicrobial agents								
Microbial isolates % (S/T)	Ampicillin	Cloxacillin	Gentamycin	Amikacin	Cefotaxime	Ceftriaxone	Pip-tazo	Meropenem	Erythromycin
Coagulase-negative Staphylococcus	91.3 (21/23)	47.8 (11/23)	82.6 (19/23)	39.13 (9/23)	56.52 (13/23)	6.25 (2/23)	26 (6/23)	17.4 (4/23)	ND
Methicillin-resistant Staphylococcus aureus (MRSA)	0 (0/1)	100 (1/1)	0(0/1)	100 (1/1)	100 (1/1)	0 (0/1)	0 (0/1)	100 (1/1)	ND
Streptococcus agalactiae	100 (2/2)	0 (0/2)	50 (1/2)	0 (0/2)	100 (2/2)	0 (0/2)	0 (0/2)	ND	ND
Bacillus spp.	100 (1/1)	100 (1/1)	100 (1/1)	100 (1/1)	100 (1/1)	0 (0/1)	100 (1/1)	ND	ND
Klebsiella spp.	50 (2/4)	100 (4/4)	100 (4/4)	100 (4/4)	50 (2/4)	25 ( 1/ 4)	ND	75 (3/4)	ND
Escherichia coli	100 (2/2)	100 (2/2)	100 (2/2)	100 (2/2)	100 (2/2)	50 (1/2)	ND	50 (1/2)	ND
Pseudomonas aeruginosa	100 (1/1)	100 (1/1)	100 (1/1)	100 (1/1)	0 (0/1)	100 (1/1)	ND	ND	ND
Enterobacter cloacae	100 (2/2)	50 (1/2)	100 (2/2)	100 (2/2)	ND	50 (1/2)	50 (1/2)	50 (1/2)	100 (2/2)
Acinetobacter baumanii	50 (1/2)	0 (0/2)	100 (2/2)	50 (1/2)	50 (1/2)	0 (0/2)	0 (0/2)	50 (1/2)	ND

## Discussion

Neonatal sepsis remains a significant health concern associated with high rates of morbidity and mortality. The initiation of appropriate empiric antimicrobial therapy is fundamental; however, it requires regular monitoring of the local epidemiological and antimicrobial sensitivity profiles of causative pathogens in order to be applied efficiently. A lack of such data may result in inappropriate use of antibiotics and the emergence of antibiotic-resistant pathogens, a major challenge in the management of sepsis. In the current study, only 16.26% of the clinically diagnosed cases of neonatal sepsis were confirmed by blood culture. This is in agreement with previous studies from other developing countries; 17.3% blood culture confirmation was reported in Ghana [1], 14% in Nepal [12], and 17.87% in North India [21]. The higher rate of inability to isolate the infectious pathogens could be attributed to small amounts of blood drawn from the infected neonates, low blood levels of the infectious agents, or maternal antimicrobial therapy prior to or during delivery. Moreover, there is a lack of consensus regarding definitions as well as substantial variation in the diagnostic criteria of neonatal sepsis worldwide; this may lead to overdiagnosis of culture-negative sepsis among neonates, therefore contributing to the higher use of antimicrobials. This is critical, as the unnecessary use of antibiotics may contribute significantly to antimicrobial resistance and negatively affect neonatal health. On the other hand, not all negative blood culture results rule out sepsis [22], and delaying prompt antimicrobial therapy raises the chances of morbidity and mortality from a potential true infection. Thus, there is an urgent need to unify the guidelines for identifying and managing culture-negative neonatal sepsis.

Our finding that the majority (67.5%, 27/40) of the isolates were gram-positive is in line with previous reports from Ethiopia [3,23], Nepal [12], India [24-25], Egypt [22], and Vietnam [26]. On the other hand, several studies have also reported the reverse (a dominant pattern of gram-negative neonatal sepsis) [22,27-33]. Isolation of CoNS, among other gram-positive bacteria, is highly frequent and accounts for >50% of cases of neonatal sepsis [22,34-36]. Consistent with previous reports, we found that 57.5% of total isolates were CoNS; this is alarming and a cause for concern as most of these isolates were known to be resistant to multiple antibiotics [34,37]. Notably, CoNS normally colonizes the skin and mucosal surfaces. However, isolation of CoNS from a sick infant with the clinical finding of sepsis most likely did not reflect the contamination of a blood culture sample, particularly considering the use of invasive medical procedures that breach the skin or mucosal barriers such as mechanical ventilators and central venous catheters [34,36,38]. Other gram-positive organisms, such as *Streptococcus agalactiae*, *Bacillus spp.*, and MRSA, have been previously reported to cause neonatal sepsis [39-41]. This would appear to indicate that most of the infections were transmitted from close contact with health care providers and relatives. In the present study, gram-negative bacteria were isolated in 26% of cases (11/40), out of which *Klebsiella spp.* predominated, at 10% (4/40). Similar findings were previously described in studies from Ghana and Nepal, in which 31% (8/26) and 13.3% (4/30) of isolates, respectively, were reported as gram-negative [1,42]. The predominant isolation of Klebsiella spp., among other gram-negative infectious pathogens, was also described in other studies [22,31-33]. However, the microbial and sensitivity profiles of pathogens causing neonatal sepsis from Saudi Arabia are very limited. One study by Al-Matary et al. reported a similar finding to those described above, in which Staphylococcus spp. were predominantly recovered from blood culture, followed by Klebsiella spp. [17]. Similarly, most of the causative bacteria of neonatal sepsis reported by Alrafiaah et al. [43] were gram-positive, with predominant isolation of CoNS being responsible for 35% (15/43) of total isolates.

Other gram-negative pathogens were also isolated in the present study. Fungal sepsis due to *Candida spp.* accounted for 5% (2/40) of culture-confirmed sepsis among neonates. Parallel findings have been reported from Saudi Arabia [43] and other developing countries [22,44-45]. In contrast to previous studies [1,5,46-48], the predominant isolates in the present study, CoNS, were highly sensitive to the first-line empiric antibiotics recommended by the World Health Organization, namely, ampicillin (91.3%) and gentamicin (82.6%). Similarly, Yadav et al. found gentamicin to be the most effective against CoNS [49]. The present findings are encouraging and require further exploration. Unexpectedly, low-to-intermediate sensitivity was observed with second and third-line empiric antibiotics, namely, cloxacillin (47.8%), amikacin (39.13%), cefotaxime (56.52%), and piperacillin + tazobactam (26%). The lower sensitivity of CoNS to cefotaxime (38%) was previously reported in Ethiopia [5]. Additionally, meropenem and ceftriaxone were associated with minimal sensitivity (17.4% and 6.25%, respectively). The increasing trend of antibiotic resistance of CoNS to various agents has been documented in multiple reports. This is significant, as not only can CoNS develop antibiotic resistance easily, but it can also transfer the developed resistance genes to other microorganisms [50-52].

In the present study, the substantial variability in the antimicrobial susceptibility pattern of first- and second-line agents could be related to the prescribing practices of physicians, which are affected by expected (rather than proven) resistance as well as antibiotic availability. This is further confirmed by the higher usage rates of meropenem as an empiric antibiotic for neonatal sepsis from low and middle-income countries [53]. Thus, accurate comparisons of antibiotic sensitivity patterns between nations are difficult. The present study was limited by its retrospective nature and scope; it had a small sample size and was conducted at a single center, KAU Hospital, which is one of many available public and private tertiary health care systems; all could serve populations with different characteristics. Although the present results cannot be generalized, the intended aim of developing an inclusive work-up of prevailing pathogenic isolates and the antibiotic susceptibility spectrum for neonatal sepsis in our institution was achieved, and this could be used to guide the proper implementation of effective empiric antimicrobial therapies.

## Conclusions

Only 16.26% of cases of neonatal sepsis in our hospital were confirmed by blood culture, with gram-positive bacteria being the most frequently isolated microorganisms. Our study highlights the possible emergence of CoNS as the main causative agent of neonatal sepsis. Infection control measures, with a particular focus on proper hand hygiene and tracing other sources as potential reservoirs for bacterial acquisition and transmission, should be carefully applied. Generally, the first-line empiric antimicrobial therapies recommended by the World Health Organization - ampicillin and gentamicin - exhibited high efficacy against most of the microorganisms isolated in this study. The unexpectedly low efficacy rates of second-line therapies require further study for clarification. For every hospital, continuous surveillance of the microbiological etiologies of neonatal sepsis and their antimicrobial sensitivity patterns must be performed for effective implementation of proper antimicrobial therapy guidelines, a potential factor in controlling antimicrobial resistance. This may help achieve not only a reduction in sepsis-related mortalities but also the use of cost-effective therapies.
